# Successful Multidisciplinary Treatment of Small Bowel Obstruction With an Ileal Stricture Resulting in Bowel Perforation in the Setting of Multidrug-Resistant Gastrointestinal Tuberculosis: A Case Report

**DOI:** 10.7759/cureus.64353

**Published:** 2024-07-11

**Authors:** Michael Ladna, David Goodson, Juliette Personius

**Affiliations:** 1 Hospital Medicine, University of California Davis Medical Center, Sacramento, USA

**Keywords:** jejunal stricture, ileal stricture, bowel perforation, small bowel obstruction, gastrointestinal tuberculosis, drug-resistant tuberculosis

## Abstract

We present the case of a male in his 40s who recently emigrated from Russia and was actively undergoing treatment for multidrug-resistant (MDR) pulmonary tuberculosis (TB) with the BPaL-M (bedaquiline, pretomanid, linezolid, moxifloxacin, and pyridoxine) regimen who presented to the emergency department (ED) with abdominal pain, vomiting, and no bowel movements. A computed tomography (CT) scan of the abdomen and pelvis revealed a small bowel obstruction (SBO) from ileal stricture consistent with gastrointestinal (GI) TB. He did not require an emergent surgical intervention and was managed conservatively via bowel rest and initiation of total parenteral nutrition (TPN). An oral BPaL-M regimen was held and an intravenous (IV) regimen consisting of linezolid, moxifloxacin, meropenem, and ampicillin/sulbactam was started per infectious disease (ID) recommendations. He improved clinically over the next several days and was started on a diet that was initially well tolerated. Shortly after transitioning to a regular diet, he developed severe abdominal pain. A CT scan of the abdomen and pelvis revealed pneumoperitoneum and he was taken emergently to the operating room (OR) for exploratory laparotomy (ex-lap). A perforation was found in the terminal ileum and he underwent a right hemicolectomy. He returned to the OR two days later for ileocolic anastomosis and fascial closure. A diet was initiated once again which was tolerated well. He was then transitioned back to his oral BPaL-M regimen which was also tolerated well. He was discharged home on an oral diet after a 23-day hospital course with follow-up appointments with acute care surgery (ACS) and ID.

## Introduction

Abdominal tuberculosis (TB) is an extrapulmonary form of TB. Gastrointestinal (GI) TB is not only difficult to diagnose given non-specific symptoms that mimic other pathologies but also because it can present without pulmonary involvement [1]. When TB involves the small or large intestines, it is called GI TB [2]. This form most commonly involves the small bowel with the ileocecal area most frequently affected and is associated with strictures and bowel obstructions [2]. Due to poor surgical outcomes in the setting of malnutrition and poor wound healing, these patients are typically managed conservatively with surgery reserved as a last resort. GI TB can be especially challenging to cure due to impairment in the absorption of oral anti-TB medications especially in the setting of an obstruction. There is a high risk of bowel perforation in GI TB patients which requires emergent surgical intervention. Due to the high mortality rate associated with primary closure, the preferred surgical intervention is resection-anastomosis [3]. Multidrug-resistant (MDR)-TB further complicates the therapeutic plan and is associated with higher mortality compared to drug-susceptible TB. Clinicians should be vigilant for GI involvement in patients with known TB but also recognize that GI TB can present without concomitant pulmonary TB [4].

## Case presentation

A male in his 40s who recently emigrated from Russia presented to the emergency department (ED) with a chief complaint of two months of progressively worsening abdominal pain and one day of vomiting of gastric contents as well as lack of bowel movement or passing gas. He was diagnosed with MDR pulmonary TB approximately two months prior for which he was prescribed the BPaL-M regimen (bedaquiline 200mg Monday, Wednesday, Friday (MWF), pretomanid 200mg daily, linezolid 900mg MWF, moxifloxacin 400mg daily, pyridoxine 100mg daily) which he reported strict compliance with. He was unsure if he had thrown up any of the tablets since vomiting started 24 hours ago before his presentation to the ED.

On examination, he was alert and orientated without acute distress. His abdomen was slightly distended and mildly tender to palpation in all quadrants without rebound or guarding. He was not actively vomiting and denied nausea. He was not encephalopathic and denied headaches, photophobia, neck pain, or neck stiffness. He was hemodynamically stable without fever, tachycardia, or hypotension.

**Figure 1:**
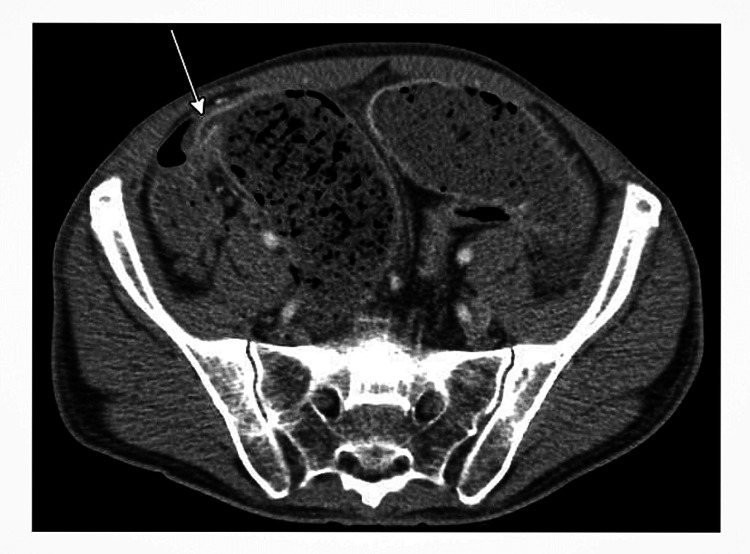
CT abdomen/pelvis with small bowel obstruction (white arrow pointing to the area of obstruction from terminal ileal stricture near the ileocecal junction).

Lab work was significant for mild leukocytosis of 12.6 109/L and microcytic anemia with hemoglobin (hgb) of 11.6 g/dL. Human immunodeficiency virus (HIV) and hepatitis C virus (HCV) were negative. A computed tomography (CT) scan of the abdomen and pelvis revealed small bowel obstruction (SBO) with terminal ileal stricture near the ileocecal junction suspicious for GI TB, focal jejunal narrowing concerning for another stricture, and small volume ascites (Figure 1). There was no radiographic evidence of closed loop obstruction due to gradual proximal transition and tapering of the bowel dilation.

The acute care surgery (ACS) team was consulted. Per ACS, emergent surgical intervention was not indicated. He was admitted to hospital medicine for conservative non-operative management. A nasogastric (NG) tube for decompression was recommended by ACS; however, the patient declined despite discussion of benefits with multiple healthcare providers. He was made strict NPO (nothing by mouth) and maintenance IV fluids for hydration were initiated. The infectious disease (ID) team was consulted. Due to the SBO and GI involvement of TB, there was concern for poor absorption of the anti-TB regimen; thus, his outpatient oral medications were held and he was started on IV linezolid 900mg MWF, IV moxifloxacin 400mg daily, and IV vitamin B6 100mg daily. He was also started on IV hydrocortisone 100mg every eight hours. Due to anticipation of an extended course of corticosteroids, he was started on pneumocystis jirovecii pneumonia (PJP) prophylaxis with IV trimethoprim (TMP)/sulfamethoxazole (SMX) 160mg daily on day 2 of hospitalization per ID recommendations. A peripherally inserted central catheter (PICC) line was placed, and he was started on total parenteral nutrition (TPN).

As the patient had a known history of TB mastoiditis, Otolaryngology (ENT) was consulted to evaluate if there was any need for surgical management of potential acute otomastoiditis. Per ENT evaluation, the patient had residual serous effusions bilaterally without evidence of acute otomastoiditis and no active otologic symptoms. There were bubbles noted behind the tympanic membrane (TM) indicating a resolving process. Since there was no evidence of acute otomastoiditis, surgical intervention was not warranted.

Gastroenterology was consulted for consideration of esophagogastroduodenoscopy (EGD) to attain a biopsy of the ileal stricture. Given the strong clinical evidence for GI TB, along with risks associated with EGD and sedation, and the clinical improvement, the decision was made to defer EGD.

On day 8 of hospitalization, the patient was started on IV meropenem and ampicillin/sulbactam was started on the day since the bedaquiline had likely washed out of his system. On day 14 of hospitalization, he began to pass gas and have bowel movements; thus, he was cleared for a full liquid diet by ACS. He tolerated the full-liquid diet and was then advanced to a regular diet.

**Figure 2:**
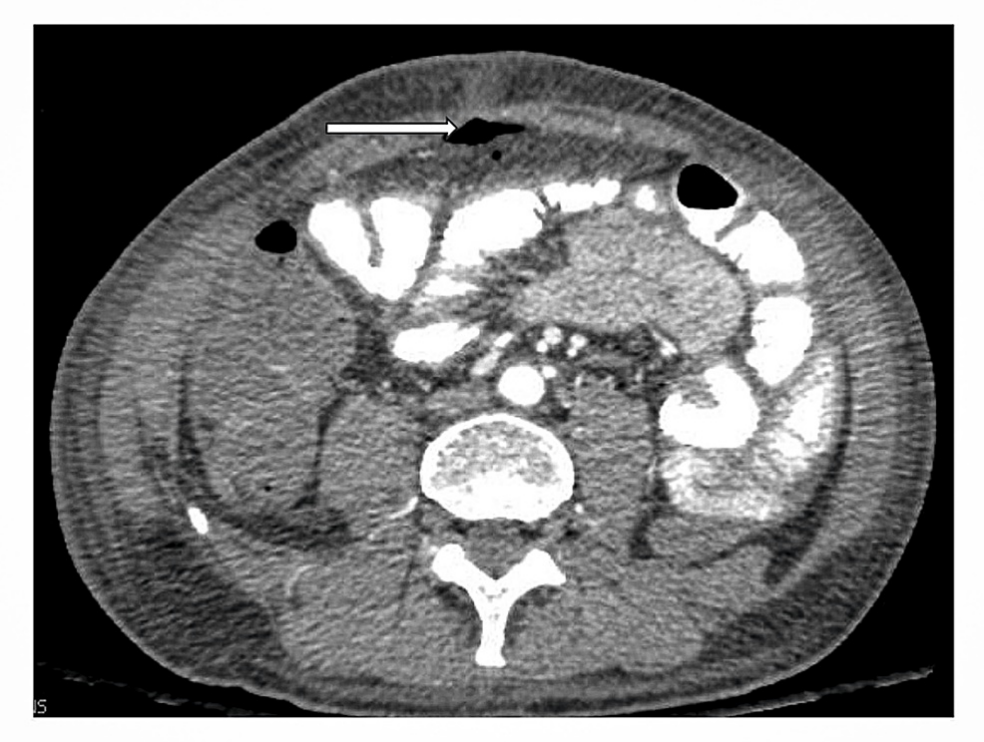
CT abdomen/pelvis with pneumoperitoneum and concern for perforation (white arrow pointing to free air in the abdomen).

On day 18 of the hospitalization, he suddenly developed severe abdominal pain. A repeat CT abdomen and pelvis showed pneumoperitoneum concerning a bowel perforation (Figure 2).

The patient was emergently taken to the operating room (OR) for exploratory laparotomy (ex-lap). During the ex-lap, a perforation was found in the terminal ileum and a right hemicolectomy was performed. He was admitted to the surgical intensive care unit (SICU) post-operatively. On postoperative day 2, he was taken back to the OR for stapling of ileocolic anastomosis and fascial closure. His pain was well controlled. The intra-operative cultures obtained grew *Clavispora lusitaniae* and *Burkholderia cepacia; *thus, he was started on IV micafungin 100mg daily on day 21 of hospitalization. He tolerated the initiation of a diet with the passage of gas and bowel movements. On day 23 of the hospitalization, his oral BPaL-M regimen was restarted; he was transitioned from IV hydrocortisone to oral prednisone 50mg daily, IV Bactrim was transitioned to oral Bactrim SS daily, and IV micafungin was transitioned to PO posaconazole 300mg BID for two days to complete treatment of peritonitis from the perforation. He tolerated all PO medications well. He attempted to leave against medical advice; however, he was persuaded to stay to coordinate his outpatient care appropriately. He was discharged after a 23-day hospitalization with plans to follow up with ID and ACS.

## Discussion

TB remains the most common cause of death by ID globally with two to three million people succumbing to the infection annually [5,6]. Risk factors associated with greater mortality include concomitant HIV infection, prior TB treatment, MDR-TB, lower body weight at the time of TB diagnosis [7], unemployment, low income, substance abuse, chronic lung disease, concomitant hepatitis infection [8], advanced age, and male sex [9]. Overall mortality rates have been decreasing with a study from the European Union showing a decrease in mortality from 5.43/100,000 and 1.37/100,000 in 2000 to 2.59/100,000 and 0.51/100,000 in 2010 for men and women, respectively [10]. Mortality remains higher in developing countries as seen in a study out of Tianjin China showing overall mortality of 4.56% [9].

GI TB accounts for 1% to 3% of all TB cases worldwide [11]. GI TB is known as the great mimicker and can be particularly challenging to diagnose given non-specific symptoms. It can mimic esophageal cancer, esophageal ulcers, gastric ulcers, gastric cancer, colorectal cancer, sarcomas, Crohn’s disease, and intestinal sarcoidosis. Accurate diagnosis is further complicated by the fact that many patients may not have concomitant active pulmonary TB. One study found that only 20% of patients with abdominal TB had associated active pulmonary TB [1].

Abdominal TB can be classified into four distinct types which are tubercular lymphadenopathy, peritoneal TB, GI TB, and visceral or solid organ TB [2]. The most frequent site of involvement is the GI tract making up 43-65% of all abdominal TB cases, followed by the peritoneum at 20-47%, lymph nodes at 4-42%, and then finally solid organs such as liver, gallbladder, spleen, and pancreas with 1-23% of cases. The small bowel is most commonly affected especially at the ileocecal which accounts for 44-84% of GI TB cases. The upper GI tract (esophagus, stomach, duodenum) is rarely involved [2].

Symptoms are typically insidious in onset and include fever, weight loss, night sweats, and anorexia. The diagnosis of abdominal TB is usually made via radiological and histopathological methods. Biopsy can be obtained via several interventions which include endoscopic mucosal biopsy, image-guided percutaneous biopsy, and surgical (open or laparoscopic biopsy). The classic histologic finding of TB is caseous granulomas.

GI TB is typically responsive to medical treatment alone with surgical intervention rarely required. Even patients who develop SBO from strictures tend to respond well to medical management alone with a prospective study of 39 patients with SBO from strictures showing that 31 (91%) had significant clinical improvement and only three (8%) failed to respond to medical therapy alone and required surgery. It should be noted that these patients did not have an MDR strain of TB and were treated with conventional anti-TB drugs (streptomycin, rifampicin, and isoniazid) [12]. Endoscopic balloon dilation is an option for patients who fail medical management and can spare the patient from surgical intervention [13]. Due to the malnourished state of this patient population, they are poor surgical candidates and thus surgery is reserved as a last resort.

Corticosteroids are used in certain cases of GI TB as they have been shown to decrease the risk of strictures and obstruction in patients with GI TB [14]. Since these patients may end up being on corticosteroids for several months, they will also require initiation of PJP prophylaxis.

Intestinal TB significantly increases the risk of bowel perforation and although emergent surgical exploration is the only curative option, post-operative mortality is especially high in this patient population. A retrospective study of 12 patients with intestinal TB who developed bowel perforation found that the most common site of perforation was the ileum (10 patients) with the other two being in the jejunum. Half of the patients (four in the ileum and both in the jejunum) had multiple sites of perforation. Seven of the patients underwent primary closure and five underwent resection-anastomosis. Three of the seven patients who had primary closure developed leaks followed by septic shock and died while all five patients who had resection-anastomosis survived [3]. Resection-anastomosis is preferred over primary closure for bowel perforation in the setting of GI TB.

Patients will require anti-TB pharmacologic treatment for at least six months, with an initial two months of isoniazid, rifampicin, pyrazinamide, and ethambutol followed by four months of rifampicin and isoniazid. Many clinicians will extend the treatment duration to 9 or 12 months.

This case was especially challenging due to the MDR profile of the TB strain. There is little data in the literature on the treatment of MDR TB involving the GI tract. Drug-resistant (DR) TB can be classified into five categories which are isoniazid-resistant TB, rifampicin-resistant (RR)-TB, MDR-TB (TB resistant to isoniazid and rifampicin), pre-extensively drug-resistant TB (pre-XDR-TB) which is MDR-TB with resistance to a fluoroquinolone and finally XDR-TB that is TB resistant to rifampicin, plus any fluoroquinolone, plus at least one further priority A drug (bedaquiline or linezolid) [15]. MDR TB is an especially difficult disease to treat and is associated with higher morbidity and mortality along with higher rates of post-TB lung damage compared to susceptible strains of TB [5]. In the United States, mortality from MDR-resistant TB has decreased from 31% during 1993-2002 to 11% in 2003-2013 [16]. Chung-Delgado et al. reported that MDR-TB had a 7.5-fold increase in mortality when compared to drug-susceptible cases [4] and the mortality of MDR-TB has been reported to be as high as 63% in patients with HIV [17].

## Conclusions

GI TB can be challenging to diagnose, in part due to non-specific symptoms that mimic other pathologies but also in that it could present without pulmonary involvement. GI TB can be especially challenging to cure due to impairment in the absorption of oral anti-TB medications. MDR-TB further complicates the therapeutic plan and is associated with higher mortality compared to drug-susceptible TB. Most of the agents in the BPaL-M regimen are not available in IV formulation. Complications from GI TB include strictures (most commonly in the ileocecal area of the small bowel), bowel obstruction, and perforation. These patients are poor surgical candidates due to often being severely malnourished and as such ensuring timely diagnosis and initiation of therapy is crucial to prevent progression to these severe complications. If emergent surgery is required, resection-anastomosis is preferred over primary closure due to better outcomes.
